# Cat Caring Behaviors and Ownership Status of Residents Enrolling a Cat in a Free Sterilization Program

**DOI:** 10.3390/ani14203022

**Published:** 2024-10-18

**Authors:** Kate Dutton-Regester, Jacquie Rand

**Affiliations:** 1 School of Veterinary Science, The University of Queensland, Gatton, QLD 4343, Australia; j.rand@uq.edu.au; 2 Australian Pet Welfare Foundation, Kenmore, QLD 4064, Australia

**Keywords:** cat ownership, semi-ownership, stray cats, cat caregiving behaviors, low socioeconomic communities, sterilization, One Welfare

## Abstract

Most cats entering animal shelters in Australia are less than 12 months old and emanate from low socioeconomic areas. Many are unidentified and may be either owned or semi-owned cats (cats cared for by someone who does not consider themselves the owner). In Australia, approximately 33% of cats entering shelters and pounds are euthanized annually, adversely affecting the mental health of staff involved. Our study investigated cat ownership and caregiving behaviors of cats enrolled in the Australian Pet Welfare Foundation’s Community Cat Program, which included free sterilization, microchipping, and preventive healthcare. The suburbs targeted in Ipswich, Australia had a high per capita cat intake into animal shelters. We surveyed 1094 cat owners and semi-owners. Most cats were under 12 months old (79%), primarily domestic short-haired (69%), and acquired through informal channels such as friends, family, or as strays. Veterinary care was limited, with only 15% of cats having visited a veterinarian and 28% being vaccinated. Prior to hearing about the free sterilization program, 88.5% of participants identified as owners, while 11.5% were semi-owners. Before completing the survey, these semi-owners transitioned from an informal caregiving relationship to becoming owner of the cat/s. Of the semi-owners, 93% indicated that their transition to ownership was driven by feelings of responsibility, emotional attachment, and access to free sterilization services. These findings suggest that targeted interventions, such as accessible veterinary care and sterilization programs, are crucial in encouraging semi-owners to take full ownership of cats, improving cat welfare, and reducing unplanned breeding. Supporting this transition helps curb overpopulation and fosters improved caregiving, benefiting both cats and communities.

## Simple Summary

Most cats entering animal shelters in Australia are less than 1 year old and originate from disadvantaged areas. Our study examined cat ownership and caregiving practices for cats being enrolled in a free sterilization program in the neighborhoods of Ipswich, Australia with high per capita intake of cats into shelters. We surveyed 1094 cat owners and semi-owners who took ownership of a stray cat at the time of sterilization, and explored caregiving practices and reasons for transition from informal caregiving to full ownership. The results revealed that most cats were under 12 months old and were primarily acquired through friends, family, or as strays. The majority of cats lacked regular veterinary care, with only a minority having visited a veterinarian or been vaccinated. Prior to hearing about the free sterilization program, 88.5% of participants identified as full owners, while 11.5% as semi-owners. These semi-owners transitioned from semi-ownership to full ownership before completing the survey. For the semi-owned cats, 93% of respondents cited a sense of responsibility, emotional attachment, and access to free sterilization services as the primary reasons for taking ownership. The findings emphasize that making veterinary care and sterilization services easily accessible is crucial for encouraging semi-owners to become full owners, which in turn improves cat welfare and reduces unwanted kittens, benefiting both cats and the community.

## 1. Introduction

Free-roaming cats in urban areas pose significant management challenges for local authorities due to their disruptive behaviors, such as urination, defecation, yowling, and fighting, which create disturbances within the community [1,2,3]. Additionally, there are concerns that free-roaming cats in urban areas pose a substantial threat to wildlife [4]. Traditional management methods, such as trapping and either adopting or euthanizing unclaimed cats, have proven ineffective and costly [5]. Further, only 5–7% of cats are reclaimed by their owner, leading to high euthanasia rates [6,7] and, subsequently, negative impacts on the mental health of shelter workers, who experience high levels of traumatic stress due to the euthanasia of healthy and treatable cats and kittens [8,9,10].

The large number of pet cats plays a significant role in the overall free-roaming cat population [11,12,13,14,15]. In Australia, approximately 33% of households own one or more cats, contributing to an estimated population of 5.3 million pet cats [13] and 0.7 million stray cats [11]. Recent efforts to mandate cat containment in Australia have not yielded the desired outcomes, resulting in increased complaints and the continued trapping and impounding of cats [5,16]. These containment measures often fail due to a lack of understanding of the root causes of the problem, particularly the socio-economic factors influencing cat ownership and management.

The best practices for cat care, including preventive health care, identification (e.g., microchipping), desexing (sterilization), and containment, are difficult to achieve where veterinary care, and the costs of containment enclosures, are unaffordable for many, and housing that provides adequate containment for cats is rare. Consequently, unplanned breeding is prevalent, leading to high numbers of free roaming cats and kittens [17,18]. A free-roaming cat in this context is a domestic cat that may be owned but not contained, a semi-owned cat that is passively acquired and is provided regular care but is not considered owned by the carer, or an unowned cat that inadvertently receives food from humans, such as from food waste bins (indirectly dependent on humans) [19,20]. Unlike in the USA, Australia defines feral cats as those that live far from human habitation and are considered pests, allowing them to be poisoned, shot, or killed with blunt force [21,22,23,24]. These feral cats are distinct from domestic cats in that they have no connection to or reliance on humans, survive through hunting or scavenging, and exist and reproduce entirely in the wild [23,24]. Because they do not inhabit areas near people, they are not typically involved in complaints about nuisance behaviors. In contrast, in the USA and many other countries, the term “feral” is also used for cats that are unsocialized or not accustomed to humans but still live in proximity to them [25,26]. These cats are often fed by well-intentioned individuals and would be classified as semi-owned domestic cats in Australia.

Free-roaming cat populations are more concentrated in low socio-economic areas [5,27,28] as is semi-ownership, or the provision of some degree of care to cat/s by people who do not consider themselves owners [17]. Free-roaming owned, semi-owned, and unowned cats are an ongoing problem, particularly in low socio-economic areas. Consequently, effective management strategies must address the specific needs of these communities where cat overpopulation is most acute. Communities facing economic challenges often struggle with limited access to affordable veterinary care, pet supplies, cat containment systems, and also may have limited access to information about the early reproductive capacity of cats and the importance of vaccinations and other preventative health care. Disadvantaged communities have higher numbers of unplanned litters of kittens due to financial hardship leading to higher numbers of cats and kittens surrendered to shelters or given away for free, and higher numbers of free-roaming, non-sterilized cats [5]. In such neighborhoods, residents might provide regular food for stray cats but are not able to provide preventive health care and do not have the ability or willingness to fully adopt them, further exacerbating the issue. Hence, targeted interventions that consider the economic constraints and provide support tailored to these communities are crucial in managing and reducing cat overpopulation and the issues they cause.

A recent study [5] reported marked decreases in complaints, impounded and euthanized cats and kittens, and costs to local governments associated with a free sterilization program targeting low socioeconomic areas with high cat-related calls and intake into the receiving animal welfare shelter and municipal pound (local government animal facility). The program focused on free sterilization, microchipping, and registration (licensing) for owned cats, and helping semi-owners take full ownership [5]. This approach not only addresses immediate animal welfare concerns but also aligns with the One Welfare philosophy, which recognizes the interconnectedness of animal, human, and environmental well-being [29,30]. From an environmental perspective, controlling the cat population helps protect local wildlife from predation, supporting biodiversity and ecosystem stability. The program benefits human well-being by reducing complaints and conflicts related to free-roaming cats, lowers costs for local governments, and enhances pet health by minimizing births of unwanted litters and the risk of infectious diseases. Furthermore, by reducing shelter overcrowding and euthanasia rates, the program alleviates the emotional toll and compassion fatigue experienced by shelter workers and volunteers, promoting job satisfaction and mental well-being [6,7,31,32,33,34,35].

To better assist cat owners and semi-owners in areas with high rates of shelter and impound admissions of cats and achieve suitable and appropriate cat management objectives, it is crucial to understand their needs more comprehensively. However, the scientific literature on owned and semi-owned cat populations in Australia, especially in low socioeconomic areas, is sparse, with limited available data. Studies with no focus on ownership or socioeconomic status report that 79% of New Zealand and Australian cats (median age of 4) had at least one veterinary visit per year and 95% were sterilized [36]. In Australia, 64% of cats received vaccinations yearly [36]. Similarly, an earlier Sydney study found that 90% of 290 cats (median age of 6) had received vaccinations, with 72% vaccinated within the past three years, and only 6% had never seen a veterinarian [37]. A national survey reported that 77% of cats were sterilized, and 72% were microchipped [38]. However, a 2012 survey of cats enrolled in a free microchipping clinic found delayed sterilization, with 93% of cats aged ≥2 years sterilized compared with 49% of cats under 2 years [39]. In contrast, among 98 semi-owned cats, 53% had never received veterinary care, and 62% had never been vaccinated [20]. Despite limited data, it is evident that cat owners and semi-owners in low socioeconomic areas represent a critical target for interventions aimed at reducing cat overpopulation, improving cat and human welfare, and lowering animal shelter intake and euthanasia rates.

In 2000, the Australian Pet Welfare Foundation (APWF) established the Community Cat Program, a five-year initiative providing free sterilization for all domestic cats in designated suburbs with high cat intakes (>20 cats/1000 residents) into the local shelters of Ipswich, Queensland, Australia. When booking the cat for surgery, participants in the Community Cat Program completed a survey containing mandatory questions regarding ownership and the cat’s health and environment, and voluntary questions related to semi-ownership. This study is novel in its focus on identifying specific factors influencing the transition from semi-ownership to full ownership, particularly within neighborhoods with high shelter intake of cats. The aim is to report on cat caring behaviors in this cohort and to explore the factors contributing to the shift from semi-ownership to full ownership.

## 2. Materials and Methods

### 2.1. Community Cat Program and Permit Approvals

The Community Cat Program is an initiative of the APWF in collaboration with the Royal Society for the Prevention of Cruelty to Animals (RSPCA) Queensland, and the Animal Welfare League Queensland. Funding and in-kind support were received from multiple organizations, including the Fondation Brigitte Bardot for sterilizations, MSD Animal Health for vaccinations and parasite control, Central Animal Records for microchip registration of cats, and Neighborhood Cats (New York, NY, USA) for cat-traps and expert advice.

As part of the Community Cat Program, cat owners and semi-owners willing to take formal ownership of the cats they were caring for by being listed as the cat’s owner on a microchip database registry were invited to complete an online booking survey for their cat’s surgery. Only owners and semi-owners willing to assume full ownership could book online. As such, all participants in the program were ultimately considered owners; however, a subset initially identified as semi-owners prior to hearing about the program and are referred to as semi-owners throughout this manuscript. These participants, along with existing owners, were asked to complete a survey as part of the booking process that consisted of both mandatory questions pertaining to the owner’s details and health of cat and voluntary questions relating to semi-ownership. The cats of all participants received free sterilization, microchipping, vaccination, parasite control, and veterinary care

Semi-owners who were unwilling or unable to take ownership but still wanted to care for the cats did not complete the survey and were managed separately under the research permit for “restricted matter”. These cats remained semi-owned and were not part of the survey-based study, as they were handled differently under the program’s research permit. The survey was only required for cats that were owned or became officially owned at the time of microchipping. The cats that remained semi-owned often required trapping and were at multi-cat sites. They were sterilized, microchipped, and ear-tipped under a research permit approved by the Queensland Government under a Department of Agriculture and Fisheries (Scientific Research Permit No. PRID000825). For the cats that remained semi-owned cats, no owner was registered on the microchip, and the cat was registered as <suburb name> Community Cat, with the Australian Pet Welfare Foundation phone number listed as the secondary contact. Human and Animal Ethics Approvals were obtained from the University of Queensland’s Research Ethics and Integrity Unit (Permit numbers Human Ethics 2019/HE002153; Animal Ethics 2019/AE000207).

### 2.2. Participant Eligibility and Recruitment

Cat owners and semi-owners in the suburbs Rosewood, Redbank Plains, and Goodna located in Ipswich, Queensland, were invited to enrol a cat in the Community Cat Program. Residents were informed about the program through advertisements on the APWF’s web and Facebook page’s (Appendix A). As the term ‘semi-owner’ is not commonly used by the general public, we invited cat owners and people feeding stray cats to contact us to organize sterilization of their cats. These suburbs were selected based on their high rates of cat shelter and impound admissions per 1000 residents in Ipswich (>20 cats/1000 residents in 2016–2017 compared with the Queensland average of 7 cats/1000 residents) [40]. There were several indicators of disadvantage in these suburbs, including Socio-Economic Indexes for Areas (SEIFA, an index measuring the relative socio-economic advantage and disadvantage of areas across Australia) scores of 847, 969, and 896, respectively, which are lower than the Ipswich local government area and the national average of 1052 and 1000, respectively. Unemployment rates across these suburbs are higher than the QLD and national averages (Table 1). Educational levels are lower than the QLD and national averages, and a larger proportion of residents have low weekly wages compared with the QLD and national averages.

In February 2023, the eligibility criteria were expanded to include additional suburbs: Bundamba, Dinmore, Ebbw Vale, Gailes, New Chum, Riverview, and surrounding areas. Interested participants were directed to BookitLive to join a waiting list. During the screening process, participants confirmed their eligibility by providing their residential address and postcode. Eligible participants received a booking link to schedule an appointment at the RSPCA Wacol Veterinary Clinic through BookitLive. Each cat required an individual booking, although one owner or carer could enrol multiple cats. During the booking process, participants were prompted to complete a survey comprising both mandatory and voluntary questions.

### 2.3. Survey Design and Distribution

The survey questions and responses used in this study (Table 2) were part of a larger, long-term study that began in 2000. The survey questions were developed with input from a panel of social scientists and psychologists. Initially, a pilot study was conducted, and responses were collected in hard copy form upon arrival at the surgery (*n* = 103). However, this method proved time-consuming, disrupted the flow of cats into surgery, and required manual data transfer to an electronic form. Therefore, the survey was reformatted as an online survey to be completed at the time that owners and semi-owners received an email with approval to book surgery (*n* = 991). An interim data check revealed that some questions were not appearing in the intended sequence (data not reported here), prompting modifications and the addition of voluntary questions regarding semi-ownership.

The present study utilizes survey data obtained between February 2022 and August 2023 and investigates the portion of the survey relating to cat caring behaviors and ownership status. Participants were asked to provide their personal details and information concerning the cat’s history, including name, sex, age, breed, and the circumstances of acquiring the cat. Additionally, they were asked to elaborate on factors potentially affecting the risk of anesthesia. Participants were also questioned about their ownership status to determine whether they considered themselves the cat’s owner or a semi-owner prior to hearing about the free sterilization program (Table 2). After this, the survey sought to elicit information pertaining to the cat’s environment and health (Table 2). The final portion of the survey consisted of a voluntary set of questions, which aimed to elicit reasons for the transition from semi ownership to full ownership (Table 2). The questions and response options used for this study are presented in Appendix B; the full survey is presented in Appendix C.

### 2.4. Data Cleaning

During the study period, there were 1177 survey responses. Of these, 53 duplicate entries were identified and subsequently removed. In resolving duplicates, the entry with the most comprehensive information or the later booking date was retained, assuming it represented the most up-to-date version. Twenty-nine respondents were identified as having come from suburbs outside of the target areas and were subsequently removed. An additional survey response was removed as the client’s information did not make sense. As a result, the final dataset consisted of 1094 unique responses. Where respondents were asked questions resulting in a numerical figure (i.e., cats age, duration of ownership), these responses were converted to months. One respondent indicated they had owned the cat ‘since kitten’; here we assumed the cat to be four weeks old when it was acquired.

### 2.5. Statistical Analysis

Data from the survey were downloaded and organized in Microsoft Excel^®^ (Version 16.89.1) spreadsheets and analyzed using ‘R’ version 2022.12.0+353 (2022.12.0+353). The age data skewed right; therefore, the median and range are reported. To examine whether there were differences between owned and semi-owned cats based on age, we used a *t*-test to compare the average ages of the two groups. For categorical variables, such as the frequency of veterinarian visits and litter tray usage, we employed the chi-square test to determine if there are significant differences between owned and semi-owned cats in these aspects. This analysis was used to understand if semi-owned cats tended to be older or younger than owned cats and if care practices differed.

## 3. Results

### 3.1. Survey Response Rate and Demographics

As all cat owners and semi-owners willing to take ownership of cats participating in the program were required to complete the survey to participate in the Community Cat Program, we achieved a 100% response rate. This included 1094 responses after data cleaning (detailed above). However, not every participant responded to every mandatory question. The response rate per question is indicated in the tables below. For the mandatory question, ‘Prior to when you heard about the free sterilization program, did you consider that you were the owner of this cat, or were you just feeding or caring for this cat?’, out of 1091 responses, 966 (88.5%) were owned cats and 125 (11.5%) were semi-owned cats. For the voluntary question, ‘When the cat first came into your life, did you regard yourself as the owner or were you just feeding or caring for this cat?’, the response rate was 71.7% (675/1094). Of these 675 respondents, 79 (11.7%) indicated that they initially cared for the cat, and 74 (94%) of these 79 respondents went on to answer additional voluntary questions regarding the factors that led to their change in ownership status. This resulted in a response rate for semi-owners for the latter two voluntary questions of 59.2% (74/125).

Of the 1094 respondents, most (66.8%; *n* = 731) were from Redbank Plains (35.4%; *n* = 387), Goodna (20.0%; *n* = 218), and Rosewood (11.5%; *n* = 126) with a combined population of 38,003 as of the last Australian Census in 2021 [41,42,44]. A further 24.1% were from Brassall (11.2%; *n* = 122), Bellbird Park (6.7%; *n* = 73), and Collingwood Park (6.2%; *n* = 68). The remaining 9% (*n* = 100) of respondents [41,42,44] were from 26 other suburbs in the Ipswich region.

### 3.2. Details of Cat Characteristics

Of the cats in the study (1094), over half (56%) were female. Most cats were ≤12 months old (79%), and the median age was 13 months (range: 2 months–9 years; Table 3). There were no statistically significant differences between owned and semi-owned cats based on age (*t* = 0.39, *p* = 0.67). Most cats (98.7%) were domestic short, medium, or long-haired cats and less than 2% were purebred (Table 3). The majority of cats were acquired from a ‘friend/family in the urban area’ (35%), via ‘online/Facebook/newspaper advertisement’ (25%), or as a ‘stray in urban area’ (14%) (Table 3).

### 3.3. Details of Environment and Preventative Health Care

Most (79.5%) respondents indicated that other cats were present in their cat’s usual environment. Almost half (46.8%) of respondents indicated that dogs were present in the cat’s usual environment; in most cases (77.5%), these dogs were vaccinated for core vaccines (canine distemper virus, canine adenovirus, and canine parvovirus). For toileting, most cats used a litter box only (76.4%), while 19.7% of cats used both a litter box and outside, but only 3.9% used outside only. Semi-owned cats were more likely to use outside only compared with owned cats (χ^2^ = 12.2, *p* = <0.01). Almost all respondents (99%) indicated that their cat has never been to a boarding facility.

Only 14.8% (*n* = 151) of respondents indicated that their cat had previously been to the veterinarian and there was no significant difference between owned and semi-owned cats (χ^2^ = 0.83, *p* = 0.44). The most common reason for a cat’s presentation to a veterinary clinic was for vaccination (46.8%) and sickness or injury (31.9%) (Table 4). Only 27.9% (*n* = 300) of cats in the study were known to be vaccinated. For those respondents who indicated that their cat was vaccinated but had not been to a veterinary clinic for vaccination (*n* = 185), the primary sources of acquisition were ‘Friend/family in the urban area’ (36.7%), ‘Online’ (23.8%), and ‘Stray in urban area (10.8%)’. The primary sources of acquisition for those who indicated that they had taken their cat to a veterinary clinic for vaccination (*n* = 55) were similar: ‘Online’ (38.2%), ‘Friend/family in the urban area’ (25.5%), and ‘Friend/family in the urban area’ (12.7%)’. Among the cats acquired as strays (both urban and rural, *n* = 115), 30.4% of respondents believed their cat was already vaccinated. Most cats had not been tested for FeLV (96%) or FIV (96%).

### 3.4. Details of Ownership Status

For the voluntary question, ‘When the cat first came into your life, did you regard yourself as the owner or were you just feeding or caring for this cat?’, of 675 respondents, 88.3% (*n* = 596) indicated that they were the owner, and 11.7% (*n* = 79) answered they were a carer for the cat. Respondents who initially did not see themselves as the cat’s owner, but indicated that they now do, were asked to choose up to 5 factors from a list of 17 that led to their change in ownership status. There were 74 respondents, 11 of which selected 6 or more options (up to 17 options were selected by some respondents); consequently, these 11 respondents were removed from the analysis of this question. For the remaining 63 respondents, the most common themes were that they wanted to do the right thing, including stopping kittens being born, they became more attached to the cat, and they heard about the availability of free sterilization (Table 5). Finally, the 74 respondents were asked which factor they considered most important; the same three themes were most common (Table 5). Overall, 93% of semi owner respondents cited being responsible/doing the right thing, increased attachment and the opportunity for free sterilization and microchipping as the primary reasons for taking ownership of their semi-owned cats. 

## 4. Discussion

This study investigated cat caregiving behaviors and cat ownership for cats being enrolled in a free sterilization, microchipping, and preventive healthcare Community Cat Program operated by the Australian Pet Welfare Foundation. It was targeted at neighborhoods of Ipswich, Australia which had a higher intake of cats into local shelters than the average for Ipswich and Queensland. Participants completed a questionnaire prior to enrolling a cat in the free cat sterilization program. The findings highlight several key points: the low rates of vaccination and veterinary visits indicate a need for accessible veterinary care; the presence of multiple cats and the prevalence of semi-ownership suggest a high potential for uncontrolled breeding; and the motivations for transitioning to ownership show that many people are willing to take responsibility for cats when supported by programs that provide free sterilization and microchipping. For these people, the transition to ownership was altruistically driven, with themes of wanting to do the right thing, feeling responsible, and growing attachment being the main factors, in addition to hearing about the free cat sterilization program. Therefore, these findings emphasize the need for targeted interventions to assist people in vulnerable communities to stop unwanted kittens from being born and improve cat welfare by providing necessary resources and support.

### 4.1. Demographics

By deliberately targeting neighborhoods with high rates of cat shelter and impound admissions, our aim was to gather a representative sample of cat owners and caregivers facing similar challenges related to access to resources and affordable veterinary care. While the majority of participants came from the primary suburbs of Redbank Plains, Goodna, and Rosewood, once we expanded the eligibility criteria to include additional suburbs, we had participants from 30 surrounding suburbs in Ipswich, indicating a broader engagement beyond our initial targets. This distribution highlights the program’s effectiveness in reaching cat owners and caregivers from various areas within the low socioeconomic stratum of Ipswich.

### 4.2. Cat Characteristics

This study revealed key insights into the characteristics of unsterilized cats being enrolled in the free sterilization program within the target communities. A significant proportion of the cats were aged ≤12 months, with almost half of these being between 2 and 6 months old, reflecting the sterilization focus of the program. These findings are consistent with the broader trend observed in many shelters, where most incoming cats are not sterilized or microchipped, are less than 12 months old, and most are under 6 months [7,45]. The high prevalence of domestic short-hair cats in the sample sheds light on the composition of feline populations in the area and suggests that they predominantly originated from the local population of randomly breeding free-roaming cats [36]. Notably, the primary sources of cat acquisition included friends or family, online platforms, newspapers, and urban strays. This reflects factors such as the unaffordability or inaccessibility of veterinary services, leading to low sterilization rates, especially in cats under two years of age. This issue is compounded by a lack of knowledge that female cats can become pregnant as early as four months old, or by the belief that a female cat should have a litter before being sterilized [46]. Additionally, high rates of free-roaming cats are promoted by factors like the scarcity of rental accommodation that allows pets or has cat-proof fencing and secure windows [46]. These conditions contribute to the unplanned and excessive breeding of both owned and unowned cats in socioeconomically disadvantaged areas. Indeed, acquiring cats from these sources often arises from their affordability, the availability of free kittens and cats, and accessibility constraints linked to traditional avenues of pet acquisition, such as pet stores, breeders, or formal adoption centers, which typically involve higher costs and associated expenses [47]. Additionally, some of these traditional sources may require the cat to be contained, which might be unattainable for some residents, particularly those who are renting. At least 16.7% of cats were passively acquired, and at least 31.1% were actively sought. Among those acquired from an urban or rural friend or family member, were given away, acquired as a kitten from an existing cat, or were ‘other’ (52.2% of cats), it is highly likely that a large proportion were acquired by the caregiver because the cat needed a home, rather than because the caregiver actively sought to acquire a cat [17]. Only 3.9% were acquired from breeders, which is consistent with reports that 6% of stray and owner-surrendered cats entering RSPCA shelters were from breeders [45].

### 4.3. Environment and Preventative Health Care

A notable finding was that about 80% of respondents reported the presence of other cats in their cat’s usual environment. This observation highlights a significant concentration of cats within low socioeconomic neighborhoods, aligning with prior research indicating that cat-related complaint calls and cat impoundments emanate at higher rates from such areas, suggesting higher densities of free-roaming cats [5,10,27,28]. This trend is likely exacerbated by low sterilization rates.

Of particular concern is the low rate of veterinary care reported by respondents, raising concern regarding overall animal welfare and the risk of diseases that would be preventable or minimized with vaccination and endo- and ectoparasite treatment. Only about 15% indicated that their cat had previously visited a veterinarian, with only about a quarter reporting at least one vaccination. In contrast, studies examining broader populations across Australia report significantly higher vaccination rates, ranging from 64% to 72% [36,37]. This discrepancy may partly stem from the predominance of cats younger than 12 months old (comprising 79% of the current study population), contrasting with other studies where only about 20% of cats fell into this age range [36,37]. However, the Australian Veterinary Association [48] recommends a core vaccination schedule comprising three doses, typically administered starting at 6–8 weeks of age and concluding by 16 weeks. The considerably lower number of respondents who reported taking their cat to a veterinarian for vaccination, compared with those who indicated their cat was vaccinated, may reflect a lack of understanding that vaccinations are typically administered by veterinarians. In some cases, there may also be misunderstandings about the cat’s vaccination status. For example, 30% of respondents who acquired their cat as a stray believed the cat was already vaccinated, despite no formal veterinary visit.

Nearly half of respondents indicated the presence of dogs in the cat’s environment, with over three-quarters of these dogs being vaccinated. This difference in vaccination rates between dogs and cats necessitates further investigation. The lower vaccination rates among surveyed cats could be due to several factors, including financial constraints, sources of acquisition, and/or educational gaps regarding cat healthcare [49,50]. Additionally, this disparity may reflect a lower willingness to pay for cat healthcare compared with dog healthcare. Data from a situational analysis conducted in the target suburbs prior to the sterilization program found that cats are less commonly acquired from breeders (12%) than dogs (36%) [31], suggesting that dog ownership is more intentional. In contrast, a significant proportion of cats (40% to 48% [22,51]) are passively acquired, meaning they are unintentionally obtained, found, or received as gifts from family or friends. This passive acquisition differs from that of dogs, with only about 24% being passively acquired, mainly through social networks or family connections [31]. Cats and dogs acquired from breeders, shelters, pounds, or rescue and foster care groups typically receive essential veterinary care such as sterilization, vaccinations, parasite treatment, and microchipping, which are included in the adoption fee. New owners also have the opportunity to discuss the animals’ needs and ongoing care. In contrast, passively acquired cats and kittens that do not go through shelters or pounds make it more challenging to ensure owners receive important information and support regarding sterilization, microchipping, and overall health and well-being.

FeLV and FIV are viral infections in cats that weaken their immune systems, making them more susceptible to other diseases [52,53]. If left untreated, these infections can be fatal due to complications arising from immune compromise [52,53]. In Australia, FeLV prevalence remains low and stable, around 0–2% [54,55]. Conversely, FIV infection exhibits a notable prevalence in owned cats, around 15% nationwide [54,56]. However, prevalence rates vary between states, ranging from as high as 20% in Western Australia to as low as 8% in South Australia [54,55]. The density of free-roaming male cats influences FIV prevalence in a population [52], suggesting a link between higher prevalence of FIV in low socioeconomic communities. Indeed, in an affluent suburb of north Queensland, prevalence was found to be 10% (*n* = 96 cats [57]; while a veterinary clinic in Melbourne catering to financially disadvantaged clients reported an FIV prevalence of 22–25% [50]. Similarly, FIV prevalence is higher in free-roaming cats from multi-cat sites in urban areas, ranging from 21 to 25% [56]. Despite these risks, most cats in our study had not been tested for FIV and were very unlikely vaccinated against it, highlighting a gap in preventive healthcare measures. We did not test clinically healthy cats for FeLV or FIV or vaccinate due to associated cost and recommendations for Community Cat Programs [58,59]. Sterilizing cats has shown to reduce the incidence of FIV, presumably because of less fighting, because FIV transmission occurs predominantly though bites [52]. Reducing fighting will also reduce the occurrence of bite-associated cellulitis and abscessation.

Limited access to veterinary services disproportionately impacts people in lower socioeconomic areas, exacerbating barriers to preventive and ongoing care for their companion animals. In many lower-income neighborhoods, the cost of veterinary care is often prohibitive for residents. Additionally, individuals without access to private transportation face significant challenges in getting their cats to a veterinary clinic because in Queensland, with the exception of pet dogs being permitted to travel on Brisbane River ferries (under certain conditions), animals are not allowed on public transport services, unless they are an approved guide, hearing, or assistance animal [60]. Many clinics close by 5 or 6 p.m., which limits accessibility, particularly for those working on casual wages who would lose income if they had to visit during the day. This lack of access results in lower rates of routine health checks, vaccinations, and treatments for common diseases. Consequently, cats in these areas are at a higher risk of contracting and spreading infectious diseases, especially FIV and panleukopenia. Additionally, the financial strain of veterinary costs can lead to delays in seeking treatment, worsening the health outcomes for affected animals. Addressing these disparities requires a multifaceted approach, including increasing the availability of affordable veterinary services, implementing community-based health initiatives, and providing information on the importance of preventive care, including sterilization, microchipping, vaccination, and parasite control.

### 4.4. Ownership Status

Semi-ownership is defined as the provision of some degree of care to cat/s by people who do not consider they own them. Previous research conducted in Australia reports semi-ownership rates ranging from 3% to 10% for regular feeders [17,20,61]. In our study, prior to the free sterilization program, 12% of respondents identified as semi-owners. These semi-owners initially regarded themselves as just feeding or caring for the cat prior to hearing about the sterilization program, but at the time of their participation in the program, they were willing to take formal ownership of the cat. Seventy-four respondents indicated that they transitioned from semi-owner to owner at some point in their relationship with their cat. This transition signifies a significant shift in individuals’ perception and commitment towards the cats under their care. Our findings highlight themes such as a sense of responsibility and wanting “to do the right thing”, deepening attachment, and the desire to prevent unwanted litters, along with the availability of free cat sterilization, as pivotal influencers of this transition. Indeed, individuals often care for cats they do not own out of compassion, sympathy, or affection for cats [19,20,62]. Furthermore, several studies report that semi-owners enjoy having the cats around and do not want the cats to be euthanized, echoing sentiments of attachment and compassion driving semi-ownership behaviors [63,64].

Overall, the desire to do the right thing, encompassing a sense of responsibility and moral obligation, emerged as the most prevalent theme. This includes factors like feeling responsible after caring for the cat, wanting to prevent the birth of kittens, and recognizing that no one else would take responsibility for the cat. These motivations reflect a conscientious approach to animal welfare, where individuals feel a duty to ensure the well-being of the cats they care for. Such motivations are crucial for understanding how semi-owners might be engaged in broader animal management and welfare initiatives [17,20].

A significant portion of respondents identified deepening attachment as a key motivator for transitioning to ownership. The prominence of deepening attachment as a primary reason for transition aligns with previous studies [20], which suggest that continued caregiving may lead to eventual ownership. The emotional bond that develops over time as individuals care for and interact with the cat reinforces their sense of commitment [19,20]. This theme is evident in responses such as “I became more attached to the cat over time”, highlighting how emotional connections grow stronger with prolonged contact [20]. Additionally, other attachment-related factors such as family members falling in love with the cat, the cat becoming more friendly, and the cat continuing to hang around further underscore the role of emotional attachment in influencing ownership decisions.

The availability of free sterilization also played a significant role in influencing ownership transition. This practical incentive reduces the financial outlay for cat carers and improves animal welfare and this aligns with the One Welfare philosophy [5,51]. The impact of free sterilization highlights the importance of accessible veterinary services in encouraging the formalization of cat ownership among semi-owners. Although less prominent, the theme of protection was also identified, with respondents expressing concerns about the cat being euthanized or harmed. These behaviors further emphasize the compassionate nature of semi-owners and their desire to ensure the safety and survival of the cats they care for [19,20,64].

Integrating a One Welfare approach into cat management strategies is essential for addressing the interconnectedness of animal, human, and environmental well-being [29,30]. One Welfare initiatives support people who have difficulty providing for their animals due to limitations in physical or mental health, income, or housing [29]. This approach extends beyond physical health to encompass ethics, economics, and politics, encouraging an interdisciplinary framework for improving human, animal, environmental, and societal welfare [29,30]. For example, community veterinary outreach helps people care for their animals, maintaining the human–animal bond while also addressing situations where both human and animal well-being are jeopardized. By reducing the intake and euthanasia of cats in shelters and pounds, these strategies not only improve animal welfare but also mitigate the negative impact on people’s mental health [6,63,65]. The stress and emotional burden associated with high euthanasia rates can significantly affect the mental well-being of shelter staff and volunteers [6,63].

However, it is important to acknowledge the potential conflicts between cat welfare and environmental well-being, particularly concerning outdoor cats. Promoting the transition from semi-ownership to full ownership through support mechanisms such as free sterilization and accessible veterinary care can lead to positive outcomes for both cats and humans. Yet, the environmental impact of outdoor cats, particularly their hunting behavior, must also be considered. There is concern that pet cats may prey on local wildlife, potentially impacting biodiversity and ecosystem balance [4,11]. Providing resources and information on protecting local wildlife is important. This involves not only encouraging full ownership but also reducing the environmental impact of cats through measures like cat containment or supervised outdoor access. In areas where managing cats can be difficult, and given concerns about wildlife predation in Australia, support could be provided to low-income communities to help install proper enclosures, window and door screens, and air conditioning, particularly in regions with threatened or endangered species. Additionally, a message to owners and semi-owners to provide their cats with a last “bed-time” meal indoors is a method that, at little or no cost to the owner, trains the cat to come inside at night when the door outside can then be closed [66]. This “bed-time” feeding can help keep “door-dasher” cats indoors at night, and train cats used to living outdoors to be inside at night, which would aid in protecting wildlife, especially since many species vulnerable to cat predation are nocturnal [10,67]. Balancing the needs of cats with the broader environmental impact is a critical aspect of the One Welfare approach.

Transitioning from semi-ownership to full ownership can impose substantial physical, financial, and emotional burdens on individuals. Many semi-owners begin caring for cats out of compassion and a sense of responsibility [3,20], but they may struggle to provide all aspects of good cat care associated with ownership. This transition can result in increased stress, financial strain, and the potential neglect of both the human’s and the cat’s needs. To address these challenges, it is essential to implement strategies that enhance cat care and welfare. Support mechanisms, such as free sterilization and accessible veterinary care, can help alleviate the financial burden on individuals, enabling them to care more effectively for their cats. However, comprehensive support is necessary to mitigate potential negative consequences. This should include integrating assistance programs and community support to benefit the health and welfare of cats, people and the environment. Specifically, these efforts should involve support for sterilization, microchipping and preventative health care, as well as encouraging cat carers to take ownership of semi-owned cats. Providing information on the early reproductive age of cats and affordable ways to keep cats contained, especially at night, is also crucial.

By elucidating the motivations behind the transition from semi-ownership to full ownership, these findings provide insights into the factors influencing people’s caregiving behaviors. Understanding these motivations enables the development of targeted interventions designed to address specific barriers faced by semi-owners, and incentives for taking ownership such as emotional attachment or concerns about unwanted litters. [29,30]. Such interventions can assist with improving cat caring practices, including sterilization and regular veterinary care, which are crucial for addressing cat overpopulation and welfare issues [5]. Consequently, insights into these motivations help in crafting more effective, tailored approaches that benefit both cats and their caregivers. Moreover, recognizing the potential role of semi-owners as key influencers or ‘gatekeepers’ [20], who can significantly impact the outcomes of community cat management initiatives, is essential. Securing their active participation and agreement (i.e., ‘buy-in’) in initiatives targeting semi-owned cat populations is critical for effective community-based cat management [17]. Building trust between municipal animal management officers and semi-owners is critical for improving the welfare of the cats and their carers [5,63]. By leveraging the knowledge, trust, and compassion of semi-owners, stakeholders (including animal welfare organizations, local authorities, veterinarians, and community groups) can develop collaborative and sustainable approaches that prioritize the welfare of both cats and their human caretakers and environmental wellbeing [5].

Furthermore, these insights underscore the importance of offering free sterilization, microchipping, and preventive healthcare where cost is a barrier to accessing these services. Creating awareness of ownership opportunities requires tailored outreach strategies targeting these communities [5]. Barriers such as accessibility need addressing, for instance, providing transportation for surgery to owners and semi-owners needing assistance, and exploring alternative booking methods beyond email and online systems to accommodate those without easy access to technology [5]. Overcoming obstacles that hinder cat owners complying with state and local government by-laws (ordinances), particularly where pet-friendly accommodation is limited, is crucial. These obstacles include local regulations like ‘cat limits’, which refer to the maximum number of cats a household is allowed to own, and ‘mandated containment’, which requires cats to be confined to their owner’s property. Mandated containment can take various forms, such as keeping cats indoors or using outdoor enclosures like large cages or fenced areas. While these regulations aim to protect both the cats and the community, they can also create significant challenges, especially for renters in properties with inadequate fencing or restrictions on modifications. For instance, the cost of effective cat containment systems, which can range from A$700 to A$2000, is prohibitive for many cat caregivers [10]. For example, in Redbank Plains, the median weekly income for a household of 3.1 people was A$1365 in 2021. Additionally, residents without air-conditioning or proper window and door screens face further difficulties in maintaining indoor containment. Therefore, addressing these financial, legislative, and practical barriers is essential to support the desired cat caring behaviors and facilitate the transition of carers to full cat owners.

### 4.5. Limitations

This study was subject to self-selection bias, which occurs when participants voluntarily choose to join a study or program group rather than being randomly selected [68]. The online booking system for the free cat sterilization program required participants to complete an online questionnaire, resulting in a 100% response rate, although not all mandatory or voluntary questions were answered. For example, for the mandatory and voluntary questions about cat ownership status, 99.1% and 61.7%, respectively, of eligible respondents answered the two similar questions. In contrast, other surveys vary in response rate from 32% to 68% [19,37,69]. While the high response rate ensured comprehensive data collection from those who participated, it did not fully eliminate the risk of self-selection bias [68].

Participation in the program depended on individuals actively seeking out and engaging with the Community Cat Program. As a result, those more engaged with animal welfare initiatives and proactive in accessing resources, such as sterilization services, may be overrepresented in our sample. Conversely, individuals facing barriers to participation—such as limited awareness of the program, lack of transportation, or other personal challenges, including no internet access—may be underrepresented.

Participation in the survey was also contingent on semi-owners transitioning to full ownership of the cat. Therefore, our study only captured the perceptions of cat owners and those semi-owners who were willing or able to be take full ownership. Semi-owners who could not or chose not to take full ownership, but still participated in the program, did not complete the survey and their cats were managed separately as “restricted matter”. These individuals often cared for multiple cats and might have different beliefs or levels of attachment compared with those who did participate. Consequently, our findings may not fully represent the broader population of semi-owners, limiting the generalizability of the results.

There is also concern that our sample may not fully represent the demographic diversity of the target population. Due to the lack of data collection on ethnicity, religion, cultural beliefs, household income, and dwelling type, we are limited in our ability to assess potential biases. Capturing these demographic variables in future studies would help us better understand and address these biases, ultimately improving the effectiveness of targeted interventions. In hindsight, including voluntary demographic questions in our survey could have provided valuable insights into the diversity of perspectives within our sample. This could guide more tailored approaches, such as translating materials into different languages or adapting messaging to cultural beliefs, to support active cat ownership in diverse communities. Information about the respondent’s family, such as the number, gender, and ages of children, as well as the presence of other cohabiting relatives like grandparents, could also have been beneficial. Future research should aim to include a more representative sample of semi-owners to better understand the barriers and motivations of those who have not transitioned to full ownership. Addressing these gaps will be crucial for enhancing the generalizability of findings and improving strategies to engage semi-owners to take ownership of the cat they are caring for.

## 5. Conclusions

This study provides valuable insights into cat caregiving behaviors and cat ownership dynamics among participants enrolling cats in a free sterilization program in suburbs with a high per capita intake of cats into the receiving shelters and municipal pound. The findings reveal significant challenges, such as low vaccination and veterinary care rates, as well as the presence of semi-owned cats. Key motivators for transitioning from semi-ownership to full ownership include a sense of responsibility and wanting “to do the right thing”, stopping kittens being born, increasing emotional attachment, and the availability of free cat sterilization and microchipping. This study underscores the importance of targeted interventions, such as accessible veterinary services and community support, to assist cat owners in effectively caring for their cats. In line with One Welfare principles, initiatives that offer free sterilization and preventive healthcare are crucial in addressing the specific needs of cat owners and semi-owners in communities with high cat pound intake per capita. This approach not only enhances animal welfare but also benefits community members’ mental well-being by reducing the stress linked to high shelter intake and euthanasia rates. However, limitations such as self-selection bias and the underrepresentation of semi-owners who have not transitioned to full ownership may affect the generalizability of the results. Future research should aim to gather more diverse demographic data and address the barriers faced by semi-owners, fostering more comprehensive and effective cat welfare initiatives.

## Figures and Tables

**Table 1:** Unemployment rate, higher education level, and median weekly household income in Rosewood [41], Redbank Plains [42], Goodna [43], Queensland (QLD), and Australia.

Suburb	Unemployment Rate (%)	Higher Education Level (%)	Median Weekly Household Income ($)	Average No. People per Household
Rosewood	10.8	6.1	1233	2.4
Redbank Plains	12.5	8	1365	3.1
Goodna	14.3	9.7	1179	2.8
QLD	7.6	18.3	1675	2.5
Australia	6.9	22	1746	2.5

**Table 2:** Survey questions answered on-line by cat owners and semi-owners at the time of booking cat/s for surgery.

**Mandatory Questions**
Prior to when you heard about the free desexing (sterilization/spay neuter) program, did you consider that you were the owner of this cat, OR were you just feeding or caring for this cat
Are there other cats present in his or her usual environment?
Did the cat come from an environment with dogs?
What are your cat’s toileting habits?
Has your cat been tested for Feline Leukemia Virus (FeLV), and if so, do you know what the result was?
Has your cat been tested for Feline AIDS (FIV), and if so, do you know what the result was?
Has your cat been vaccinated?
Was a full-course (two or more) of vaccinations given as a kitten?
If vaccinated, when was the last vaccine given?
Has this cat been to a veterinarian before for any reason
What did they go to the veterinarian for?)
**Voluntary questions**
When the cat first came into your life, did you regard yourself as the owner or were you just feeding or caring for this cat?
If at first you didn’t consider yourself to be the owner, but now you do, what factors made you change your mind?
Among the factors you selected which was the most important?

**Table 3:** Characteristics of cats included in the study including age, breed, and cat acquisition source.

**Cat’s Age (*n* = 1042)**	**% (*n*)**
2–≤6 months	49.1% (512)
>6 months–≤12 months	29.9% (312)
>1–≤3 years	16.4% (171)
>3–9 years	4.5% (47)
**Cat’s Breed (*n* = 964)**	
Domestic short hair	68.6% (661)
Domestic medium hair	22.4% (216)
Domestic long hair	7.7% (74)
Purebred	1.3% (13)
**Where did your cat originally come from? (*n* = 1078)**	
Friend/family in the urban area	34.5% (372)
Online/Facebook/newspaper advertisement	25.2% (272)
Stray in urban area	13.7% (148)
Friend/family in rural area	9.4% (101)
Kitten from existing cat	5.5% (59)
Breeder	3.9% (42)
Stray in rural area	3.0% (32)
Giveaway- owner didn’t want	1.4% (15)
Other	1.4% (15)
Animal shelter/pound	1.3% (14)
Pet shop	0.7% (8)

**Table 4:** Vaccination details relating to the number of vaccines administered and recency of vaccination for 114 of the 300 cats said to be vaccinated, and reasons for 141 cats’ presentation to a veterinarian clinic.

Questions Relating to Vaccinations and Veterinary Visits	% of All Responses (n)
**Was a full course (two or more) of vaccinations given as a kitten? (*n* = 114)**	
Yes	83.3% (95)
No	9.7% (11)
Don’t know	7.0% (8)
**If vaccinated, when was the last vaccine given? (*n* = 187)**	
Within the last 6 months	67.9% (127)
6–12 months ago	17.6% (33)
Over a year ago	12.3% (23)
1–3 years ago	2.1% (4)
**What did they go to the veterinarian for? (*n* = 141)**	
Sick/injury	31.9% (45)
Vaccination only	30.5% (43)
Other	17.0% (24)
Vaccination and other	16.3% (23)
Microchip only	4.3% (6)

**Table 5:** Factors influencing change in ownership perception. Main themes were R = wanted to do the right thing; A = became more attached; S = free sterilization; P = protect; O = other.

**If at first you didn’t consider yourself to be the owner, but now you do, what factors made you change your mind—select up to 5 that apply; Among the factors you selected which was the most important—select only one from the list below).**	**Top 5 ^1^; % of All Responses (*n*)**	**Most** **Important ^2^;** **% of All** **Responses (*n*)**	**Theme/s**
I wanted to do the right thing	54.0 (34)	25.7% (19)	R
To stop kittens being born	47.6 (30)	18.9% (14)	R
I became more attached to the cat over time	46.0 (29)	14.9% (11)	A
I feel responsible now because I have been caring for it	42.9 (27)	12.2% (9)	R
I heard about the opportunity to get cat sterilized ^3^ & microchipped for free	33.3 (21)	12.2% (9)	S
I was concerned this cat would be euthanised	23.8 (15)	4.1% (3)	P
The kids or another family member fell in love with it	20.6 (13)	2.7% (2)	A
There is no one else to take responsibility for this cat	20.6 (13)	2.7% (2)	R
The cat was at risk of being trapped by animal control or council officers	14.3 (9)	1.4% (1)	P
Other	14.3 (9)	1.4% (1)	-
Initially I was not sure if this cat belonged to someone	12.7 (8)	1.4% (1)	O
The cat continued to hang around	11.1 (7)	1.4% (1)	A
The cat became more friendly	9.5 (6)	1.4% (1)	A
The cat started coming into the house	7.9 (5)	0 (0)	A
The cat was in danger of being hurt	6.3 (4)	0 (0)	P
I was not sure someone else was feeding and caring for this cat	3.2 (2)	0 (0)	O
The cat required veterinary attention	1.6 (1)	0 (0)	R/P

Participants were asked: ^1^ If at first you didn’t consider yourself to be the owner, but now you do, what factors made you change your mind—select up to 5 that apply; and ^2^ Among the factors you selected which was the most important (select only one from the list below). There were 63 and 74 responses, respectively; ^3^ the term desexing instead of sterilization or spay neuter was used in the survey because that is more commonly understood in Australia.

**Figure A1:**
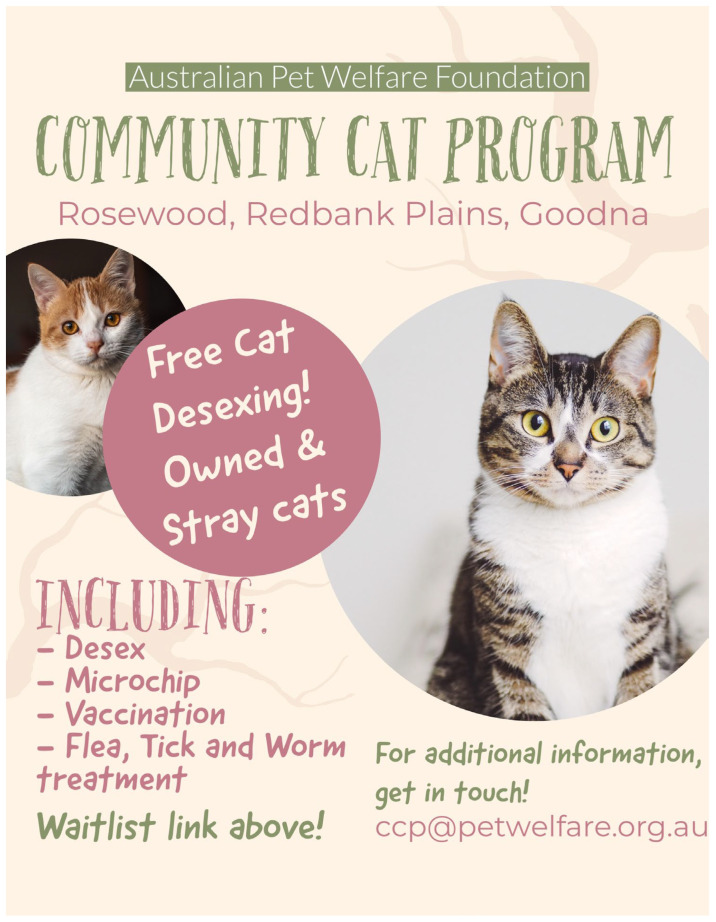
Example advertisement posted on Facebook to recruit participants for the Community Cat Program free sterilization initiative.

## Data Availability

Most relevant data are reproduced in the text. Full data are available upon reasonable request.

## Appendix B

**Table A1:** The subset of questions from the full survey that were used in the present study.

Survey Questions	Response Options
**Mandatory survey questions**	
Prior to when you heard about the free desexing (sterilization/spay neuter) program, did you consider that you were the owner of this cat, OR were you just feeding or caring for this cat	Owner/Just feeding or caring for cat
2. Are there other cats present in his or her usual environment?	No, single cat household/Yes, multi-cat household
3. Did the cat come from an environment with dogs?	Yes/No/Unknown
4. Are those dogs vaccinated?	Yes/No/Unknown
5. Where did your cat originally come from?	Animal shelter or pound/Friend or family in urban area/Stray in urban area/Stray in rural area/Breeder/Friend or family in rural area/Online, Facebook, newspaper advertisement/Other
6. What are your cat’s toileting habits?	Litter-box only/Both litter-box and outside/Outside only
7. Does your cat ever go to boarding facilities?	Yes/No
8. Has your cat been tested for Feline Leukemia Virus and if so, do you know what the result was?	Not tested/Positive/Negative/Don’t know
9. Has your cat been tested for Feline AIDS, and if so, do you know what the result was?	Not tested/Positive/Negative/Don’t know
10. Has your cat been vaccinated?	Vaccinated/Unvaccinated/Unknown
11. Was a full-course (two or more) of vaccinations given as a kitten?	Yes/No/Don’t know
12. If vaccinated, when was the last vaccine given?	Within the last 6 months/6–12 months ago/1–3 years ago/Over 3 years ago
**Voluntary survey questions**	
13. When the cat first came into your life, did you regard yourself as the owner or were you just feeding or caring for this cat	Owner/Just feeding or caring for cat
14. If at first you didn’t consider yourself to be the owner, but now you do, what factors made you change your mind (select up to 5 that apply) 15. Among the factors you selected which was the most important (select only one from the list)?	I became more attached to the cat over time I heard about the opportunity to get cat desexed (sterilized) and microchipped for free The cat continued to hang around The cat became more friendly I wanted to do the right thing The cat was at risk of being trapped by animal control or council officers I was concerned this cat would be euthanized To stop kittens being born I feel responsible now because I have been caring for it There is no one else to take responsibility for this cat Initially I was not sure if this cat belonged to someone I was not sure someone else was feeding and caring for this cat The cat started coming into the house The kids or another family member fell in love with it The cat was in danger of being hurt The cat required veterinary attention Other (please explain)

## Appendix C

**Table A2:** The full survey required to be completed by respondents when booking a cat for free sterilization and microchipping in the Community Cat Program.

Survey Question	Response Options
**I. Your cat**	
1. How long have you owned or cared for this cat?	
2. Prior to when you heard about the free desexing program, did you consider that you were the owner of this cat, OR were you just feeding or caring for this cat?	Owner/Just feeding or caring for cat
**II. Your cat’s environment**	
1. How would you describe your cat’s outdoor access?	Indoors only/More outdoors than indoors/More indoors than outdoors/Outdoors only/50/50
2. What is the extent of your cat’s outdoor access?	Enclosed courtyard or enclosed yard/Not restricted to my property boundaries/NA
3. When is your cat able to have access to the outdoors?	Day-time only/Night-time only/Day- and night-time/NA
4. Are there other cats present her usual environment?	No, single-cat household/Yes, multi-cat household/How many other cats?
5. Did the cat come from an environment with dogs?	Yes/No
6. Are those dogs vaccinated?	Yes/No/Unknown/NA
7. Where did your cat originally come from?	Animal shelter or pound/Friend or family in urban area/Stray in urban area/Stray in rural area/Breeder/Friend or family in rural area/Online, Facebook, newspaper advertisement/Other
8. What are your cat’s toileting habits?	Litter-box only/Both litter-box and outside/Outside only
9. Does your cat ever go to boarding facilities?	Yes/No
**III. Your cat’s health and vaccination status**	
10. Has your cat been tested for Feline Leukemia Virus (FeLV), and if so, do you know what the result was?	Not tested/Positive/Negative/Don’t know
11. Has your cat been tested for Feline AIDS (FIV), and if so, do you know what the result was?	Not tested/Positive/Negative/Don’t know
12. Has your cat been vaccinated?	Vaccinated/Unvaccinated/Unknown
13. Was a full-course (two or more) of vaccinations given as a kitten?	Yes/No/Don’t know
14. If vaccinated, when was the last vaccine given?	Within the last 6 months/6–12 months ago/1–3 years ago/Over 3 years ago
**IV. Your cat’s diet**	
15. Which statements (tick all that apply) best describes your cat’s diet?	All wet food from cans or pouches/All dry food/A combination of commercial wet and dry food/A combination of raw meat and commercial food/A combination of cooked meat and commercial food
16. Do you ever feed your cat raw meat?	Yes/No
17. If yes, how often?	Daily/Weekly/Monthly/Occasionally
18. What types (tick all that apply) of raw meat have you fed your cat?	Chicken/Beef/Lamb/Pork/Rabbit/Kangaroo/Other (Please specify)
19. Does your cat hunt?	Yes, often/Sometimes/Never
20. If yes, have you ever seen your cat eat the prey?	Yes/No
21. What prey (tick all that apply) have you seen your cat catch?	Insects/Slugs and snails/Lizards & geckos/Rodents (mice, rats)/Birds/Other
On average, how many insects does your cat catch in 6 months?	
On average, how many slugs and snails does your cat catch in 6 months?	
On average, how many lizards and geckos does your cat catch in 6 months?	
On average, how many rodents (mice, rats) does your cat catch in 6 months?	
On average, how many birds does your cat catch in 6 months?	
On average, how many ‘other’ prey does your cat catch in 6 months?	
**This question is voluntary to answer, but the information will help us better help people feeding cats that need desexing (sterilizing)**	
22. When the cat first came into your life, did you regard yourself as the owner or were you just feeding or caring for	Owner/Just feeding or caring for cat
23. If at first you didn’t consider yourself to be the owner, but now you do, what factors made you change your mind (select up to 5 that apply from the list below)? 24. Among the factors you selected which was the most important (select only one from the list)?	I became more attached to the cat over time I heard about the opportunity to get cat desexed (sterilized) and microchipped for free The cat continued to hang around The cat became more friendly I wanted to do the right thing The cat was at risk of being trapped by animal control or council officers I was concerned this cat would be euthanized To stop kittens being born I feel responsible now because I have been caring for it There is no one else to take responsibility for this cat Initially I was not sure if this cat belonged to someone I was not sure someone else was feeding and caring for this cat The cat started coming into the house The kids or another family member fell in love with it The cat was in danger of being hurt The cat required veterinary attention Other (please explain)
